# ^18^F-FDG PET/CT Metrics Are Correlated to the Pathological Response in Esophageal Cancer Patients Treated With Induction Chemotherapy Followed by Neoadjuvant Chemo-Radiotherapy

**DOI:** 10.3389/fonc.2020.599907

**Published:** 2020-11-27

**Authors:** Nicola Simoni, Gabriella Rossi, Giulio Benetti, Michele Zuffante, Renato Micera, Michele Pavarana, Stefania Guariglia, Emanuele Zivelonghi, Valentina Mengardo, Jacopo Weindelmayer, Simone Giacopuzzi, Giovanni de Manzoni, Carlo Cavedon, Renzo Mazzarotto

**Affiliations:** ^1^ Department of Radiation Oncology, University of Verona Hospital Trust, Verona, Italy; ^2^ Department of Medical Physics, University of Verona Hospital Trust, Verona, Italy; ^3^ Department of Nuclear Medicine, University of Verona Hospital Trust, Verona, Italy; ^4^ Department of Oncology, University of Verona Hospital Trust, Verona, Italy; ^5^ Department of General and Upper G.I. Surgery, University of Verona Hospital Trust, Verona, Italy

**Keywords:** positron emission tomography metrics, pathological response, induction chemotherapy, chemo-radiation, esophageal cancer, radiomic features, neoadjuvant therapy

## Abstract

**Background and Objective:**

The aim of this study was to assess the ability of Fluorodeoxyglucose Positron Emission Tomography/Computed Tomography (^18^F-FDG PET/CT) to provide functional information useful in predicting pathological response to an intensive neoadjuvant chemo-radiotherapy (nCRT) protocol for both esophageal squamous cell carcinoma (SCC) and adenocarcinoma (ADC) patients.

**Material and Methods:**

Esophageal carcinoma (EC) patients, treated in our Center between 2014 and 2018, were retrospectively reviewed. The nCRT protocol schedule consisted of an induction phase of weekly administered docetaxel, cisplatin, and 5-fluorouracil (TCF) for 3 weeks, followed by a concomitant phase of weekly TCF for 5 weeks with concurrent radiotherapy (50–50.4 Gy in 25–28 fractions). Three ^18^F-FDG PET/CT scans were performed: before (PET_1_) and after (PET_2_) induction chemotherapy (IC), and prior to surgery (PET_3_). Correlation between PET parameters [maximum and mean standardized uptake value (SUV_max_ and SUV_mean_), metabolic tumor volume (MTV), and total lesion glycolysis (TLG)], radiomic features and tumor regression grade (TGR) was investigated.

**Results:**

Fifty-four patients (35 ADC, 19 SCC; 48 cT3/4; 52 cN+) were eligible for the analysis. Pathological response to nCRT was classified as major (TRG1-2, 41/54, 75.9%) or non-response (TRG3-4, 13/54, 24.1%). A major response was statistically correlated with SCC subtype (p = 0.02) and smaller tumor length (p = 0.03). MTV and TLG measured prior to IC (PET_1_) were correlated to TRG1-2 response (p = 0.02 and p = 0.02, respectively). After IC (PET_2_), SUV_mean_ and TLG correlated with major response (p = 0.03 and p = 0.04, respectively). No significance was detected when relative changes of metabolic parameters between PET_1_ and PET_2_ were evaluated. At textural quantitative analysis, three independent radiomic features extracted from PET_1_ images ([JointEnergy and InverseDifferenceNormalized of GLCM and LowGrayLevelZoneEmphasis of GLSZM) were statistically correlated with major response (p < 0.0002).

**Conclusions:**

^18^F-FDG PET/CT traditional metrics and textural features seem to predict pathologic response (TRG) in EC patients treated with induction chemotherapy followed by neoadjuvant chemo-radiotherapy. Further investigations are necessary in order to obtain a reliable predictive model to be used in the clinical practice.

## Introduction

Esophageal Cancer (EC) is a major health problem worldwide, representing the 7^th^ leading cause of cancer-related mortality (1). In locally advanced stage disease, a preoperative approach (chemotherapy or chemo-radiotherapy) is currently accepted as standard of care (2). In particular, randomized trials evaluating neoadjuvant chemo-radiotherapy (nCRT) followed by surgery, have demonstrated a 10%–15% improvement in long-term survival rate with trimodality therapy as compared with surgery alone (3–5). Notably, Tumor Regression Grade (TRG) of the primary tumor after nCRT is a well-established prognostic factor to predict long-term prognosis in EC (6–8). Hence, in order to identify a subset of patients who would most likely benefit from nCRT, the availability of prognostic and predictive markers for response, is strongly advocated.

In our experience, after the completion of a phase II study, an intensive nCRT protocol consisting of induction chemotherapy (IC), followed by concurrent chemo-radiotherapy (CRT), and thereafter by surgery, was considered the standard approach for both Squamous Cell Carcinoma (SCC) and Adenocarcinoma (ADC) of the esophagus and gastroesophageal junction (EGJ). In our series, 5-year survival rates were 77% for pathological complete response (pCR), 44% for near pCR (microfoci of tumor cells on the primary tumor), and 14% for residual tumor subsets, respectively (p < 0.001) (9). It can be hypothesized that the use of induction chemotherapy may allow to screen patients with EC in “good responders”, in which CRT following IC may determine an effective survival advantage and therefore should be used, and in “bad responders”, in which CRT could be unnecessary or even detrimental due to possible adverse events. In this unfavorable group, surgery should not be further delayed or, alternatively, a change in systemic therapy should be adopted.

Fluorodeoxyglucose Positron Emission Tomography/Computed Tomography (^18^F-FDG PET/CT), combining functional PET information with anatomical CT images, is routinely used for diagnosis, radiation treatment planning, and response evaluation in various gastrointestinal malignancies (10–13). In particular, the role of ^18^F-FDG PET/CT for baseline staging, restaging before surgery, and recurrence/distant metastases detection during follow-up in EC is well established (14, 15). Furthermore, it represents a useful, non-invasive tool to assess the response to neoadjuvant chemotherapy and chemo-radiotherapy. Several traditional PET parameters, such as maximum and mean standardized uptake value (SUVmax and SUVmean), metabolic tumor volume (MTV), and total lesion glycolysis (TLG), have demonstrated the ability to provide functional information useful in predicting pathological response to nCRT in EC patients (16–18). More recently, radiomic features are emerging as promising tools to stratify patients in “good” or “bad” responders. Some studies have investigated different first, second and high-order features, in EC patients, demonstrating that PET radiomic parameters can predict the response to nCRT (19–25). In addition, Chen et al. postulated that using a combination of traditional and radiomic PET parameters can provide a better stratification of patients into different prognostic subgroups (26).

Based on this background, we performed a novel analysis to evaluate the ability of ^18^F-FDG PET/CT metrics to predict histological tumor regression in patients with EC treated with an intensive nCRT protocol.

## Material and Methods

### Study Design

This is a single-center retrospective analysis of prospectively collected data, approved by the Institutional Review Board of our Hospital. Inclusion criteria were: a) patients treated with an intensive nCRT protocol for locally advanced resectable SCC or ADC of the esophagus or EGJ (Siewert I and II); b) availability of ^18^F-FDG PET/CT scans performed before and after induction chemotherapy, and before surgery; c) surgical resection; d) availability of resection specimens for pathological analysis. Exclusion criteria were: a) chemo-radiation therapy approaches other than the nCRT protocol (e.g., CROSS scheme); b) Siewert III type (candidates for peri-operative chemotherapy) and SC cervical tumors (treated with definitive CRT); c) upfront resectable (cT1 or cT2N0) or metastatic disease; d) non-execution of at least one of the three ^18^F-FDG PET/CT scans; e) no surgical resection; f) unavailability of resection specimens.

### nCRT Protocol and Surgery

**Figure 1:**
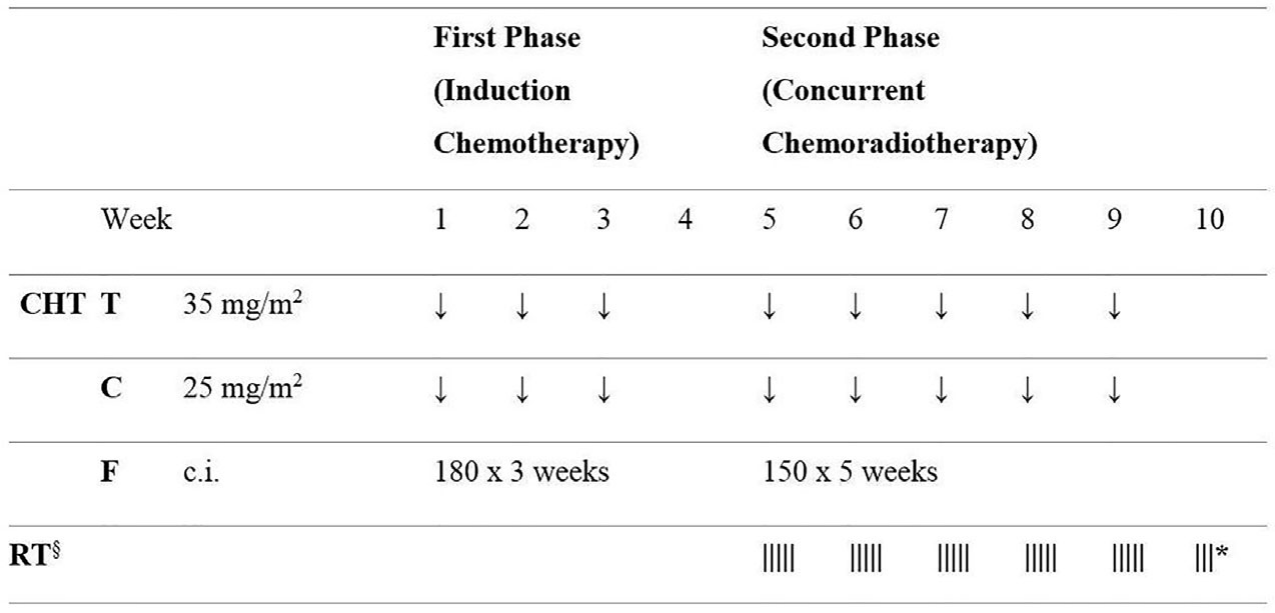
Schematic diagram of the neoadjuvant chemo-radiotherapy protocol schedule. CHT, chemotherapy; T, docetaxel; C, cisplatin; F, 5 fluorouracil; c.i., continuous infusion; RT, radiotherapy. Doses of 5 fluorouracil (F) are given as mg/m^2^/day. ^§^ RT 50-50.4 Gy in 25–28 fractions; * if 28 RT fractions are used.

The nCRT protocol consisted of a first phase of induction chemotherapy with docetaxel, cisplatin and 5-fluorouracil (TCF) for 3 weeks (days 1-22), followed by a second phase of concurrent chemotherapy (TCF) and radiotherapy for 5–6 weeks (days 29-63), as previously described (9). Radiation therapy (RT) was delivered with volumetric modulated arc therapy (VMAT), prescribing 50-50.4 Gy in 25-28 fractions. **Figure 1** describes the nCRT protocol schedule. Sample VMAT plans are presented in **Figures S1** and **S2** (**Supplementary Material**).

After restaging, surgery with radical intent was performed 8 weeks after nCRT completion. Tri-incisional subtotal esophagectomy (McKeown procedure), partial esophagectomy (Ivor-Lewis procedure) or total gastrectomy with distal esophagectomy was performed based on tumor characteristics.

### ^18^F-FDG PET/CT Method and Metrics

**Figure 2:**
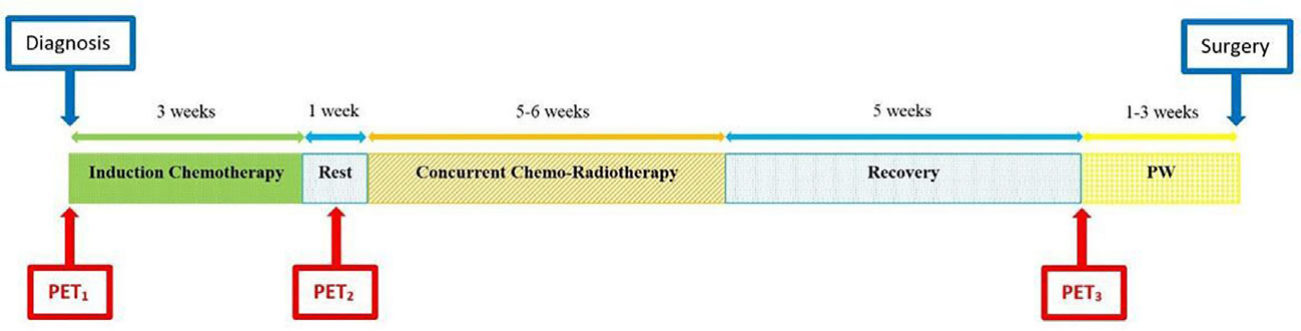
Diagram of total neoadjuvant protocol from diagnosis to surgery, including induction chemotherapy and concomitant chemo-radiotherapy, with ^18^F-FDG PET/CTs at relative time points. PW, preoperative workup, including restaging.

Three ^18^F-FDG PET/CT scans were performed: the first (PET_1_) at baseline, before the start of the induction phase and the second (PET_2_) before the concomitant phase (week 4 of nCRT protocol). PET_1_ and PET_2_ were performed with the patient in RT treatment position, as simulation for volume delineation and treatment planning. The third ^18^F-FDG PET/CT (PET_3_) was performed during restaging prior to surgery. **Figure 2** shows ^18^F-FDG PET/CT relative time points.

The ^18^F-FDG PET/CT scan was performed using the Gemini TF Big Bore system (Philips Medical Systems, Eindhoven, The Netherlands) at our Nuclear Medicine Department. All patients were asked to fast for at least 6 h and blood glucose levels were checked before imaging. Patients underwent a whole-body scan, from skull base to mid-thigh, starting 60 ± 10 min after the intravenous injection of 3 MBq/Kg of ^18^F-FDG. The acquisition parameters for diagnostic CT scan were: 120 kV, 60–80 mAs, pitch 0.813, collimation 16x1,5 mm, field of view (FOV) 600 mm. CT scan images were reconstructed using a filtered back projection with 5 mm thickness and 512x512 matrix. For simulation, CT mAs automatic modulation and 3 mm thickness reconstruction were adopted. The acquisition time of PET scanning was 1.15 min per bed position, with a FOV of 576 mm. PET images were reconstructed using list mode ordered subset expectation maximization (LMOSEM) algorithm (144 x 144 matrix, 4 mm/pixel, 4 mm slice thickness). CT images were used to correct the PET emission data for photon attenuation.

For this analysis, the tumor was segmented on the ^18^F-FDG PET/CTs dataset using a semi-automatic gradient-based method called “PET Edge” (MIM software, Mim Software Inc., US), which identifies the boundary of the metabolically active tumor based on the surface defined by the maximum gradient of metabolic activity. Quantitative parameters were extracted from the ^18^F-FDG PET/CT scans at the three time points (PET_1_, PET_2_, and PET_3_) previously reported. These parameters were: maximum and mean standardized uptake value (SUV_max_ and SUV_mean_), metabolic tumor volume (MTV) and total lesion glycolysis (TLG), defined as the product of MTV and SUV_mean_.

### Radiomic Feature Extraction

Radiomic features were extracted from PET_1_ and PET_2_ without applying any gray-level normalization nor voxel resampling. Indeed, according to the IBSI guidelines, calibrated gray levels should not be further standardized and the cubic voxel spacing was the same for the whole dataset. The DICOM files (volumes and RT Structures) were converted to the nii format through dcmrtstruct2nii (27). Since the gradient-based contouring algorithm, used to define the VOI, tends to exclude the most peripheral zones of the lesion, with the consequent inclusion of a limited number of voxels, the VOIs were dilated by 1 voxel (4 mm) in each direction. This was performed by using the built-in BinaryDilateImageFilter method of SimpleITK (v1.2.4) (28), implemented in Python (v3.7.6) under conda (v4.8.2) environment. In addition to increasing the number of voxels included in the lesion, dilating the VOI also allows the analysis to be performed on the low-enhancement region of the tumor, potentially adding information on how the uptake decreases on the lesion boundary. Radiomic features were extracted through pyradiomics (v3.0), an open-source python package (29). All the available features implemented in pyradiomics were extracted: Shape, First Order, GLCM, GLDM, GLRLM, GLSZM, and NGTDM [the meaning of these acronyms can be found in Zwanenburg et al. (30)]. For gray-level discretization, a fixed bin-count of 64 bins was adopted. A hundred and five features were extracted from both PET_1_ and PET_2_.

### Pathological Analysis

Postsurgical pathology examination provided macroscopic and microscopic description of the primary tumor and retrieved nodes. Post-resection staging was assessed following ypTNM categories according to the International Union against Cancer (UICC, 7^th^ edition, 2010). The degree of pathologic response was scored using the tumor regression grade (TRG) classification according to a modified Mandard score system: TRG1 = no residual cancer cells; TRG2 = residual cancer cells scattered through fibrosis; TRG3 = increased residual cancer cells with predominant fibrosis and TRG4 = including TRG4, residual cancer predominant fibrosis, and TRG5, no regressive changes within the tumor, of the Mandard score system (6). Patients were grouped according to the pathological response to nCRT in two classes of outcome. Major pathologic response was defined as TRG1–2 while non-response as TRG3–4.

### Statistical Analysis

The association between clinical/radiomic data and response to treatment was first analyzed by means of the chi-square test or Fisher’s exact test for categorical parameters, and Student’s T-test for continuous quantities (age and length of tumor). The association between PET/CT metabolic parameters and response to treatment (defined as a dichotomous variable) was assessed by means of logistic regression and ROC analysis. For logistic regression analysis, quantitative metabolic parameters were logarithmically transformed to meet the assumption of linearity on the logit scale, as in van Rossum et al. (18). Quantitative variables were described as median and interquartile range (IQR) or mean and standard deviation (SD), and categorical variables were summarized as counts and percentages. Statistical analysis was performed using R version 4.0.2 (https://www.R-project.org/) and MATLAB version R2019a (The Mathworks, Inc.; Natick, Massachusetts, USA). The significance level of the radiomic analysis was computed at p < 0.05/N, where N = 210 is the number of the tested features considering both PET_1_ and PET_2_ (Bonferroni correction). Two features were considered strongly correlated (*i.e.* redundant) when the pairwise Pearson’s correlation coefficient was higher than 0.90; in this case, the feature with the highest average correlation with all the other features was removed. The significance level for all tests was assumed at p < 0.05.

## Results

### Study Population

Ninety-eight patients with biopsy-proven locally advanced esophageal squamous cell carcinoma (SCC) or adenocarcinoma (ADC), who underwent neoadjuvant chemo-radiotherapy at our Institution between January 2014 and December 2018, were retrospectively identified. Forty-four patients were excluded from the study for the following reasons: preoperative approach other than nCRT protocol (n=31); no surgery (n=8); lack of at least one ^18^F-FDG PET/CT scan (n=5). The remaining 54 patients were considered eligible for analysis. Among them, 41 (75.9%) showed a major pathologic response (TRG1-2) and 13 (24.1%) a non-response (TRG3-4) to nCRT.

All patients completed the nCRT planned program. PET_1_ was performed immediately before (median 8.5 days, IQR 6–14) the start of IC, while PET_2_ was performed during the 4th week (median 25 days, IQR 22–28) of the nCRT protocol schedule. PET_3_ was performed 5 weeks (median 5.1 weeks, IQR 4.0-5.4), and surgery 8 weeks (median 7.9 weeks, IQR 6.6–9.1) after nCRT completion.

At the last follow-up, 33 patients (61.1%) were alive. The median follow-up of the entire cohort was 32.5 months (IQR 26.0–45.0 months). The median overall survival (OS) and disease-free survival (DFS) of the TRG1-2 group at the time of last follow-up were 34.7 months (IQR 27.5–49.7 months) and 30.7 months (IQR 17.1–47.7 months), respectively, while the corresponding figures for the TRG3-4 group were 28.0 months (IQR 23.4–30.8 months) and 18.1 months (IQR 10.4–30.7 months), respectively (p < 0.01).

### TRG and Baseline Characteristics

**Table 1** reports the results of the analysis of the association between baseline parameters and response to the nCRT protocol. Parameters that statistically correlated to outcome were histological subtype (p = 0.02) and tumor length (p = 0.03). All other parameters evaluated were not statistically linked to treatment outcome.

**Table 1:** Baseline features with significance of association to treatment outcome.

	Major response(n=41)	Non-response(n=13)	*p* value^§^
**Male gender**	33 (80.5%)	11 (84.6%)	0.74
Age (years)**	64.3 ± 8.9	59.9 ± 8.9	0.12
**Tumor location**			0.10
Medial	15 (36.6%)	1 (7.7%)	
Distal	12 (29.3%)	4 (30.8%)	
EGJ	14 (34.1%)	8 (61.5%)	
**Histological subtype**			0.02*
SCC	18 (43.9%)	1 (7.7%)	
ADK	23 (56.1%)	12 (92.3%)	
Length of tumor (cm)**	5.4 ± 2.1	6.8 ± 1.5	0.03*
**Clinical T stage**			0.90
T1/T2	5 (12.2%)	1 (7.7%)	
T3	33 (80.5%)	11 (84.6%)	
T4	3 (7.3%)	1 (7.7%)	
**Clinical N stage**			—
N0	2 (4.9%)	0 (0.0%)	
N+	39 (95.1%)	13 (100.0%)	

^§^p-value of chi-square test, Fischer’s exact test or Student’s T-test.

*statistically significant.

**mean ± SD.

### TRG and Metabolic Parameters

**Figure 3:**
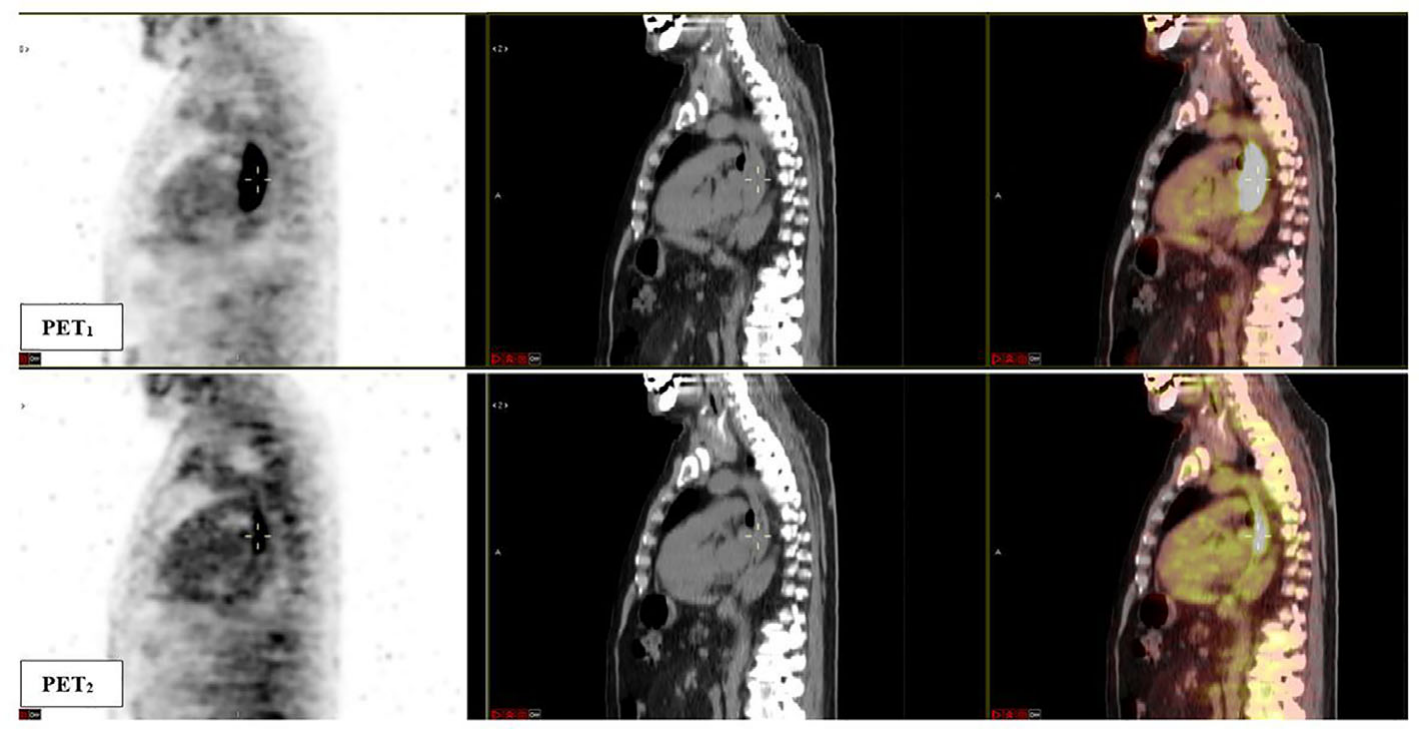
Sagittal fused ^18^F-FDG PET/CT images obtained at baseline (PET_1_) and after induction chemotherapy (PET_2_). A significant response to induction chemotherapy (reduction in metabolic parameters) of the esophageal lesion can be observed. The patient was classified as TRG1 at final pathological examination. PET_1_ parameters: SUV_max_ 26.9, SUV_mean_ 12.7, MTV 43.7 ml, TLG 553.9; PET_2_ parameters: SUV_max_ 6.7, SUV_mean_ 3.2, MTV 15.9 ml, TLG 50.5.

**Figure 4:**
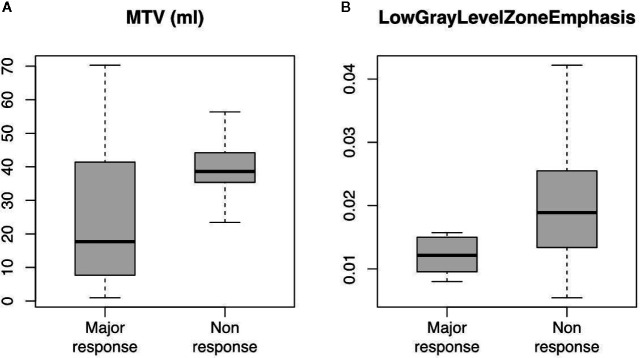
Boxplot distribution of **(A)** MTV (ml) and **(B)** LowGrayLevelZoneEmphasis (GLCM) radiomic feature at ^18^F-FDG PET/CT scan taken before induction chemotherapy (PET_1_). Classes are divided between major (TRG1-2) and non (TRG3-4) response (median, interquartile and full range are displayed).

**Table 2** reports the results of the analysis of the association between metabolic parameters of PET_1_ and PET_2_ with tumor regression grade class (TRG1-2 vs. TRG3-4). Relative differences between the parameters at the two subsequent PET/CT scans are also reported. At baseline, MTV (AUC 0.74) and TLG (AUC 0.69) were statistically correlated to histological tumor regression. In addition, at PET_2,_ SUVmean (AUC 0.67) and TLG (AUC 0.64) were significantly related to a higher chance of major pathologic response (**Figure 3**). No significance was detected when relative differences were considered. Interestingly, none of the post-CRT PET metrics resulted significantly correlated with the outcome measured (average SUV_max_ was 5.01 vs. 5.09, SUV_mean_ 2.81 vs. 2.75, and MTV 8.74 ml vs. 8.74 ml, in TRG1-2 vs. TRG3-4 patients, respectively; all p > 0.05). Therefore, additional analysis, relative to PET_3_ metrics, was not conducted. **Figure 4A** reports the boxplot distribution of the MTV at PET_1_, the parameter most correlated to treatment outcome at logistic regression analysis.

**Table 2:** Results of the logistic regression and ROC curve analysis of metabolic 18F-FDG parameters before and after induction chemotherapy, with their relative differences.

	Major response median (IQR)	Non-response median (IQR)	OR (95% C.I.)	p value	AUC
**SUV_max_**					
PET_1_	13.3 (9.3, 16.1)	13.3 (10.5, 15.1)	0.69 (0.02 - 22.66)	0.84	0.44
PET_2_	5.8 (4.5, 7.2)	6.6 (6.3, 9.8)	0.03 (0.00 - 1.08)	0.05	0.65
PET_1_-PET_2_ relative difference [Δ SUV_max_ (%)]	-43.3 (-65.9, -24.7)	-40.3 (-52.8, -24.4)	0.98 (0.96 – 1.01)	0.20	0.56
**SUV_mean_**					
PET_1_	6.1 (5.1, 7.1)	6.2 (4.6, 8.6)	1.55 (0.03 - 93.97)	0.83	0.43
PET_2_	3.1 (2.5, 4.0)	3.7 (3.4, 5.0)	0.01 (0.00 – 0.59)	0.03*	0.67
PET_1_-PET_2_ relative difference [Δ SUV_mean_ (%)]	-40.8 (-59.1, -29.4)	-38.6 (-44.2, -14.6)	0.98 (0.96 – 1.01)	0.19	0.54
**MTV (mL)**					
PET_1_	17.7 (7.7, 41.4)	38.6 (35.4, 44.2)	0.03 (0.00 - 0.51)	0.02*	0.74
PET_2_	10.8 (6.6, 16.2)	13.9 (10.8, 19.3)	0.15 (0.02 – 1.41)	0.10	0.62
PET_1_-PET_2_ relative difference [D MTV (%)]	-44.2 (-72.4, -22.4)	-63.6 (-70.8, -55.6)	1.02 (0.99 – 1.04)	0.15	0.62
**TLG**					
PET_1_	112.3 (54.1, 265.5)	216.8 (178.3, 300.8)	0.07 (0.01 – 0.63)	0.02*	0.69
PET_2_	30.2 (18.1, 61.5)	51.1 (30.8, 93.5)	0.11 (0.01 – 0.94)	0.04*	0.64
PET_1_-PET_2_ relative difference [Δ TLG (%)]	-72.7 (-88.0, -48.8)	-73.2 (-86.6, -62.7)	1.01 (0.99 – 1.03)	0.34	0.47

*statistically significant.

### Radiomic Feature Analysis

**Figure 5:**
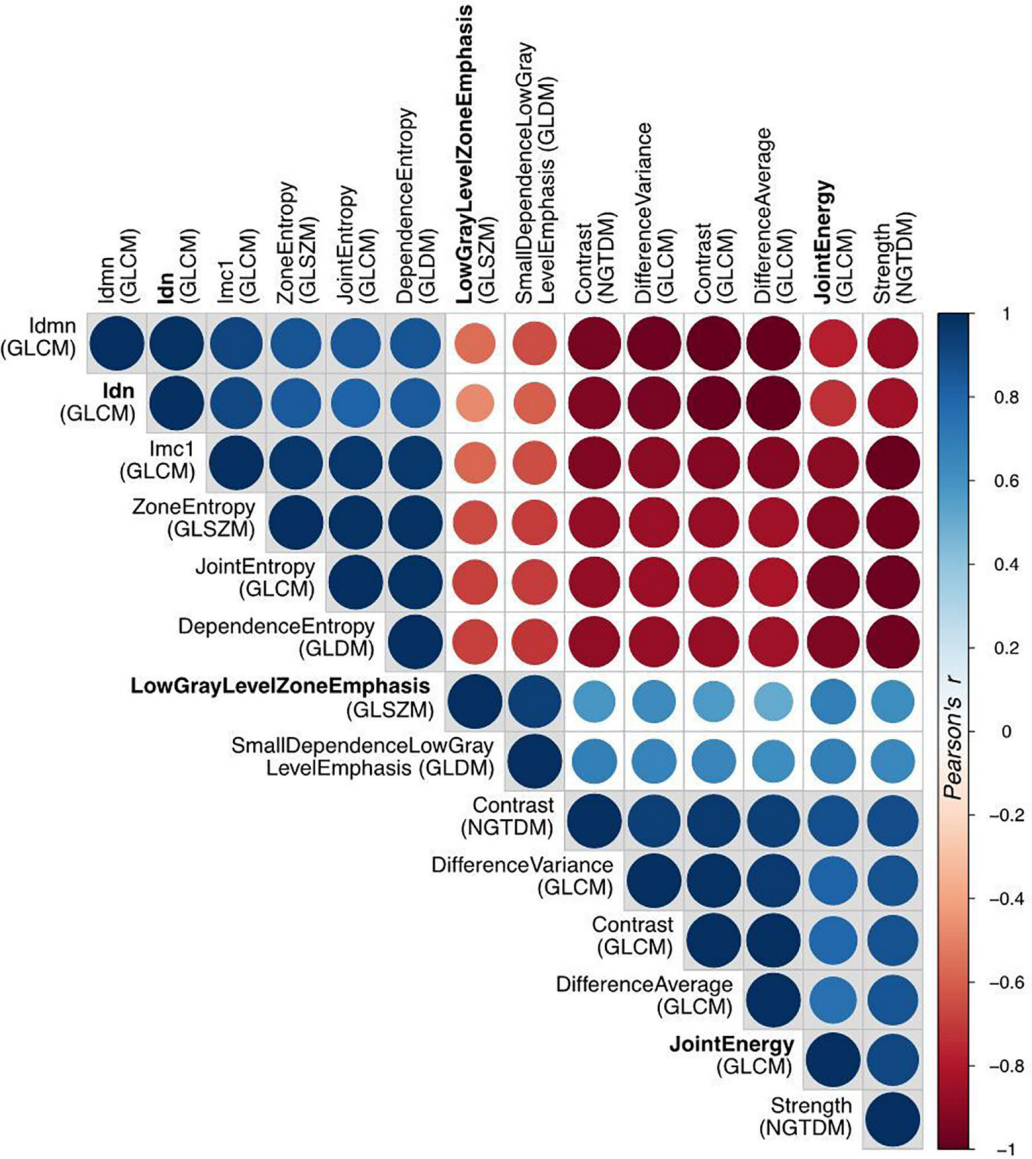
Correlation matrix of all the radiomic features with high significance (p<0.0002) in t-test for patient response. The Pearson’s correlation coefficient identifies three disjointed clusters, in which the three representatives (Idn=InverseDifferenceNormalized, JointEnergy and LowGrayLevelZoneEmphasis) are highlighted in bold.

**Figure 6:**
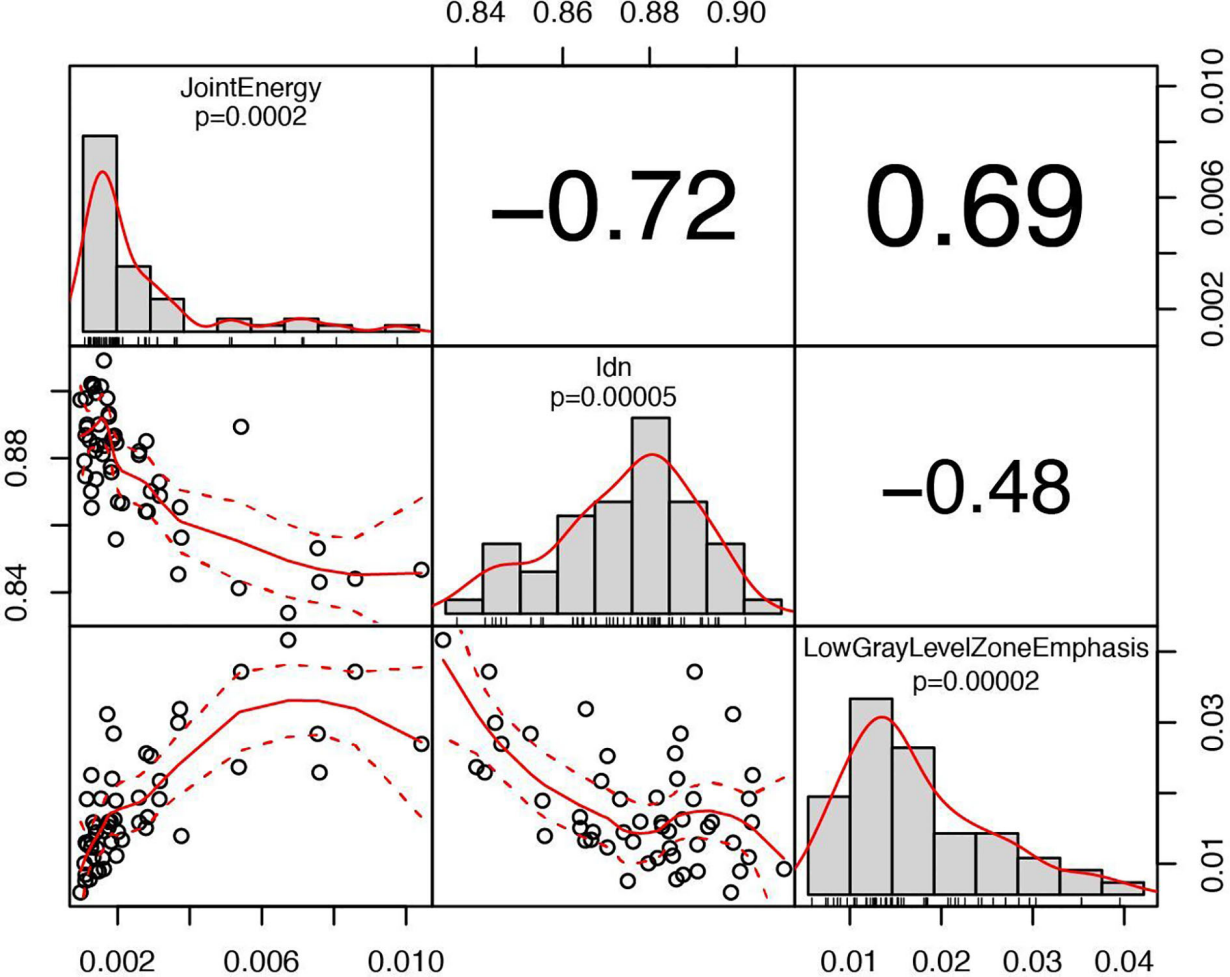
Correlation chart between the selected radiomic features with a high significance for predicting patient response. The name of the feature, the significance level and the distribution are displayed on the diagonal. In the top triangular part, the absolute value of the Pearson’s correlation coefficient is reported. In the bottom, the bivariate scatterplot is visible together with the fitted line according to a second order local polynomial regression.

Among the 210 radiomic features (105 for each PET scan), 14 resulted significant to the t-test with the adjusted significance threshold p_Th_ = 0.05/210 = 0.00024 and none of them were extracted from the PET_2_ scan. Since many of these features are strongly correlated, as visible in **Figure 5**, the redundant information was removed resulting in three independent features. The three resulting features, highlighted in **Figure 5** with bold fonts, are representative for the whole cluster and further reported in **Figure 6** with the relative scatterplots and histograms. The boxplot of one of these three features is reported in **Figure 4B** (LowGrayLevelZoneEmphasis).

## Discussion

Neoadjuvant chemo-radiotherapy (nCRT) has been widely accepted as the standard of care for the treatment of locally advanced, resectable esophageal cancer. However, a not negligible number of patients show a poor response to neoadjuvant therapy at the time of surgery (residual tumor on the resection specimen), as an expression of pre-existing intrinsic chemo- and radio-resistance. Notably, non-responder patients to nCRT have a significantly worse prognosis than responders (6–8, 31). Thus, there is an urgent need to early identify patients who could benefit or not from preoperative treatment, using prognostic and predictive tumor biology markers. This study demonstrated that ^18^F-FDG PET/CT metrics may be able to predict the degree of pathologic response, according to a modified Mandard tumor regression grade (TRG) score system, in patients undergoing induction chemotherapy (IC) followed by chemo-radiotherapy as an intensive neoadjuvant protocol.

Metabolic parameters, that were statistically correlated to treatment outcome, were the MTV (p = 0.02) at PET_1_, and SUV_mean_ (p = 0.03) at PET_2_. The TLG was also significant at both time-points (p = 0.02 and p = 0.04, respectively). This can be interpreted as a consequence of the above, as TLG is defined as the product between MTV and SUV_mean_. These results might suggest that the lesion volume, as determined before IC, and the average metabolic activity, as determined after IC, should be considered significant. In addition, at textural quantitative analysis, three independent radiomic features extracted from PET_1_ images (JointEnergy and InverseDifferenceNormalized of GLCM and LowGrayLevelZoneEmphasis of GLSZM) were statistically correlated with major response (p < 0.0002). This indication could be important in view of a possible early prediction of outcome, with potential advantages to patients believed to benefit from the three-stage treatment. However, further investigations are necessary in order to obtain a reliable predictive model to be used in the clinical practice, especially if the inclusion of radiomic features in the model is foreseen. In fact, additional validation is mandatory in the latter case since predictive models, based on radiomics, are more prone to overfitting compared to models based on conventional PET parameters.

The results of the present study could lead to different considerations. Metabolic tumor volume (MTV) combines the information of SUV uptake and tumor volume, corresponding to the volume of tumor tissues with increased glycolytic activity. In our study, MTV in poor responders was significantly higher than in good responders (38.6 ml vs. 17.7 ml, p = 0.02). Since SUV_max_ and SUV_mean_ did not differ significantly between the two groups (TRG1-2 vs. 3-4) at PET_1_, this figure appears to be consensual to the greater extent of the primary tumor in non-responder patients at the time of diagnosis (tumor length 6.8 cm vs. 5.4 cm in good responders, p = 0.02). This result is consistent with previous experiences reported in the literature, showing a link between tumor length and outcome in EC patients (32, 33). This could suggest that the MTV of the primary tumor has the potential to become a valuable prognostic biomarker for response at baseline in EC patients. The other baseline parameter, significantly correlated with TRG class after nCRT, was squamous histological subtype (p = 0.02). This confirms the greater sensitivity to nCRT of the squamous histology compared to ADC, as reported in the literature (34). In this regard, whether surgery on demand is advisable in selected complete responder SCC patients after nCRT is currently under evaluation in the randomized SANO trial (35).

At PET_2_ evaluation, SUV_mean_ represents the main predictor for pathological response (p = 0.03). The SUV_mean_ provides information about intrinsic lesion characteristics, related to tumor grading, biological factors, and the presence of hypoxic or necrotic areas. Thus, it is reasonable to hypothesize that SUV_mean_ after IC might be an effective predictor of the final response to nCRT. This post-induction chemotherapy assessment could help to guide a PET-adapted preoperative strategy in EC. Recently, a Memorial Sloan Kettering Cancer Center series tested the impact of changing concurrent chemotherapy, during radiotherapy, in PET non-responders after IC: no survival benefit was seen from this change in therapeutic strategy (36). On the other hand, in the MUNICON trial, after 2 weeks of IC, ^18^F-FDG PET/CT poor responders were referred to immediate surgery, while good responders continued with preoperative chemo-radiotherapy (37). The results of this trial suggested no decrement in survival outcomes with early termination of ineffective chemotherapy in PET non-responders, supporting a possible early discontinuation of preoperative treatment in this subset of patients. In the near future, the integration of ^18^F-FDG PET/CT metabolic parameters, magnetic resonance imaging (MRI) data, and genomic and molecular information (e.g. liquid biopsies), could lead to a more individualized treatment approach for non-responder patients (38).

The value of metabolic parameters, to predict response to nCRT in EC, has been obtained from heterogeneous studies with remarkable differences in the adopted protocols and outcomes measured. Possible predictive valuable metrics are: the percentage decrease in TLG (36), the SUV_max_ (39), the percentage decrease in MTV and TLG (40), the MTV and TLG at PET performed on day 21 of nCRT (41), or the relative changes in MTV and SUV_mean_ at PET performed after 11 fractions of RT (42). Differently from other authors (36, 42), we did not observe any significant correlation with the metabolic parameters measured when relative differences were evaluated. Therefore, considering the aforementioned differences between the present and other studies, the role of ^18^F-FDG PET/CT traditional metrics as a predictor of response is undoubtedly intriguing, but requires further investigation.

The analysis of radiomic features revealed that textural characteristics of PET_1_ were more significantly correlated to treatment response compared to PET_2_, confirming the possible predictive value of PET_1_. Many radiomic features were correlated to each other, suggesting a redundancy of information that should be carefully taken into account if using radiomics in a predictive model. To this regard, extensive validation is necessary. However, the textural metrics, that correlate to treatment outcome, are associated to micro-variations of local metabolic activity thus indicating a possible role of spatial intra-tumor heterogeneity in predicting response (19). Relatively few studies have investigated the role of PET radiomic features in predicting response to nCRT in EC. As a whole, the results have highlighted a possible contribution of radiomic in the prognostic stratification of these patients (**Table 3**).

**Table 3:** Recent findings on the application of PET radiomics for the prediction of response in esophageal cancer patients treated with neoadjuvant chemo-radiotherapy (summary).

Study, year (ref)	Sample size	nCRT protocol	PET time point	Main features evaluated	Results
Tixier et al. (19)	41 (ADC 10, SCC 31)	60 Gy + C or carboplatin/F	Pre-CRT	First order statistics GLCM RLM GLSZM NGTDM	Tumor textural analysis (GLCM homogeneity, GLCM entropy, RLM intensity variability and GLSZM size zone variability) can identify NR, PR and CR with higher sensitivity (76%–92%) than any SUV measurement
Tan et al. (20)	20 (ADC 17, SCC 3)	50.4 Gy + C/F	Pre & Post-CRT	First order statistics GLCM	SUV_max_ decline, SUV_mean_ decline and skewness, GLCM inertia, correlation and cluster prominence, are predictors of CR (AUC 0.76–0.85)
Van Rossum et al. (21)	217 ADC	45–50.4 Gy + fluoropyrimidine/platinum or taxane	Pre & Post-CRT	First order statistics Geometry GLCM NGTDM	At multivariate analysis baseline cluster shade, Δrun percentage, ΔICM entropy, and post-CRT roundness, correlates with CR
Yip et al. (22)	45 (ADC 44, SCC 1)	45–50.4 Gy + C, F, irinotecan/paclitaxel or carboplatin/paclitaxel	Pre & Post-CRT	GLCM: homogeneity, entropy RLM: high gray run emphasis, short-run high gray run emphasis GLSZM: high gray zone emphasis, short-zone high-gray emphasis	Change in run length and size zone matrix parameters differentiate CR/PR from NR (AUC 0.71–0.76)
Beukinga et al. (23)	97 (ADC 88, SCC 9)	41.4 Gy + carboplatin/paclitaxel	Pre-CRT	First order statistics Geometry GLCM NGTDM	Long runs (coarse texture) with low gray levels and homogeneity of runs (fine texture) higher in patients with CR
Nakajo et al. (24)	52 SCC	41–70 Gy + C/F	Pre-CRT	GLCM: Entropy, homogeneity, dissimilarity; GLSZM: Intensity variability, Size-zone variability, zone percentage	Texture features (GLSZM intensity variability and GLSZM size-zone variability), and volumetric parameters (MTV and TLG) can predict tumor response
Beukinga et al. (25)	70 (ADC 65, SCC 8)	41.4 Gy + carboplatin/paclitaxel	Pre & Post-CRT	First order statistics Geometry Local intensity GLCM GLSZM NGTDM	The combination of clinical T-stage and post-nCRT joint maximum predict CR
Chen et al. (26)	44 SCC	50 Gy + platinum-based regimen	Pre-CRT	SUV variance, standard deviation, skewness, kurtosis, and entopy NGLCM TFCCM NGTDM	Pre-CRT primary tumor histogram entropy ≥ 3.69 predicts unfavorable response

nCRT, neoadjuvant chemo-radiotherapy; C, cisplatin; F, 5 fluorouracil; NR, non response; PR, partial response; CR, complete response.

The predictive value of post-CRT ^18^F-FDG PET/CT functional informations has been largely evaluated, with conflicting results. Indeed, the utility of PET metrics after radiotherapy remains controversial due to difficulties in distinguishing post-treatment inflammation from residual viable tumor (43, 44). In the present study, none of the PET_3_ metrics resulted significantly correlated with the pathological response to nCRT, confirming that metabolic parameters relative to post-CRT PET are poorly evaluable and potentially inaccurate, mostly due to post-radiation inflammatory-related uptake or the disappearance of detectable metabolic activity, both considered as confounding factors. On the other hand, the use of ^18^F-FDG PET/CT imaging before surgery, in appropriate combination with other restaging modalities, remains essential for the early detection of loco-regional and distant progression to nCRT.

Our study presents some limitations, including its retrospective nature. This is a single-center analysis; thus the results should be interpreted with caution. Moreover, different histologies (SCC and ADC) were considered, which potentially add heterogeneity to the outcomes measured. On the other hand, these limitations are counterbalanced by the analysis of one of the most homogeneous sample sizes for this topic so far, with patients undergoing the same intensive nCRT protocol, using a prospectively collected database, a standardized ^18^F-FDG PET/CT acquisition modality and a modern metabolic and radiomic parameter analysis.

In conclusion, our observations confirm that ^18^F-FDG PET/CT metrics are correlated with pathological response in EC. The analysis of PET traditional metrics and radiomic features may provide a new imaging perspective, moving from tumor staging to a promising role in disease stratification and prognostication. However, further studies are needed to justify a PET-guided strategy in the neoadjuvant approach to locally advanced EC. The integration with MRI data, as well as genomic and molecular analysis, might be useful as prognostic and predictive biomarkers for the selection of a tailored strategy improving the efficiency of neoadjuvant treatment for EC patients.

## Data Availability Statement

The datasets presented in this article are not readily available because the datasets generated for this study are available on request to the corresponding author, subject to approval by the Institutional Review Board. Requests to access the datasets should be directed to NS, nicola.simoni@aovr.veneto.it.

## Ethics Statement

The studies involving human participants were reviewed and approved by Comitato etico per la Sperimentazione Clinica (CESC) delle Province di Verona e Rovigo. Written informed consent for participation was not required for this study in accordance with the national legislation and the institutional requirements.

## Author Contributions

Conceptualization, NS and CC. Methodology, NS, GB, and CC. Data curation, MZ, RMi, MP, EZ, and VM. Statistical analysis and radiomics, GR, GB, and CC. Validation, SGu, JW, and SGi. Writing—original draft preparation, NS, MZ, and CC. Writing—review and editing, GR, RMi, JW, and SGi. Supervision, GD and RMa. All authors contributed to the article and approved the submitted version.

## Conflict of Interest

The authors declare that the research was conducted in the absence of any commercial or financial relationships that could be construed as a potential conflict of interest.

The handling editor declared a past co-authorship with one of the authors with several of the authors [NS, RMa].
