# Supplementary material for: 18F-FDG PET/CT Metrics Are Correlated to the Pathological Response in Esophageal Cancer Patients Treated With Induction Chemotherapy Followed by Neoadjuvant Chemo-Radiotherapy
Source: Front Oncol. 2020 Nov 27;10:599907. doi: 10.3389/fonc.2020.599907 (PMC7729075; doi:10.3389/fonc.2020.599907)

**Figure S1.** Sample plan for an esophageal cancer patient (Siewert II, adenocarcinoma, cT3N1) treated with volumetric modulated arc therapy (VMAT). (A) Axial, (B) coronal, (C) sagittal views of the dose distribution, target volumes and organs at risk (OARs). (D) Dose-volume histogram (DVH). The following structures are shown: planning target volume (red); clinical target volume (cyan); gross tumor volume (lemon green); heart (orange); lungs (white); liver (brown); spinal cord (yellow); and bowel (light green).


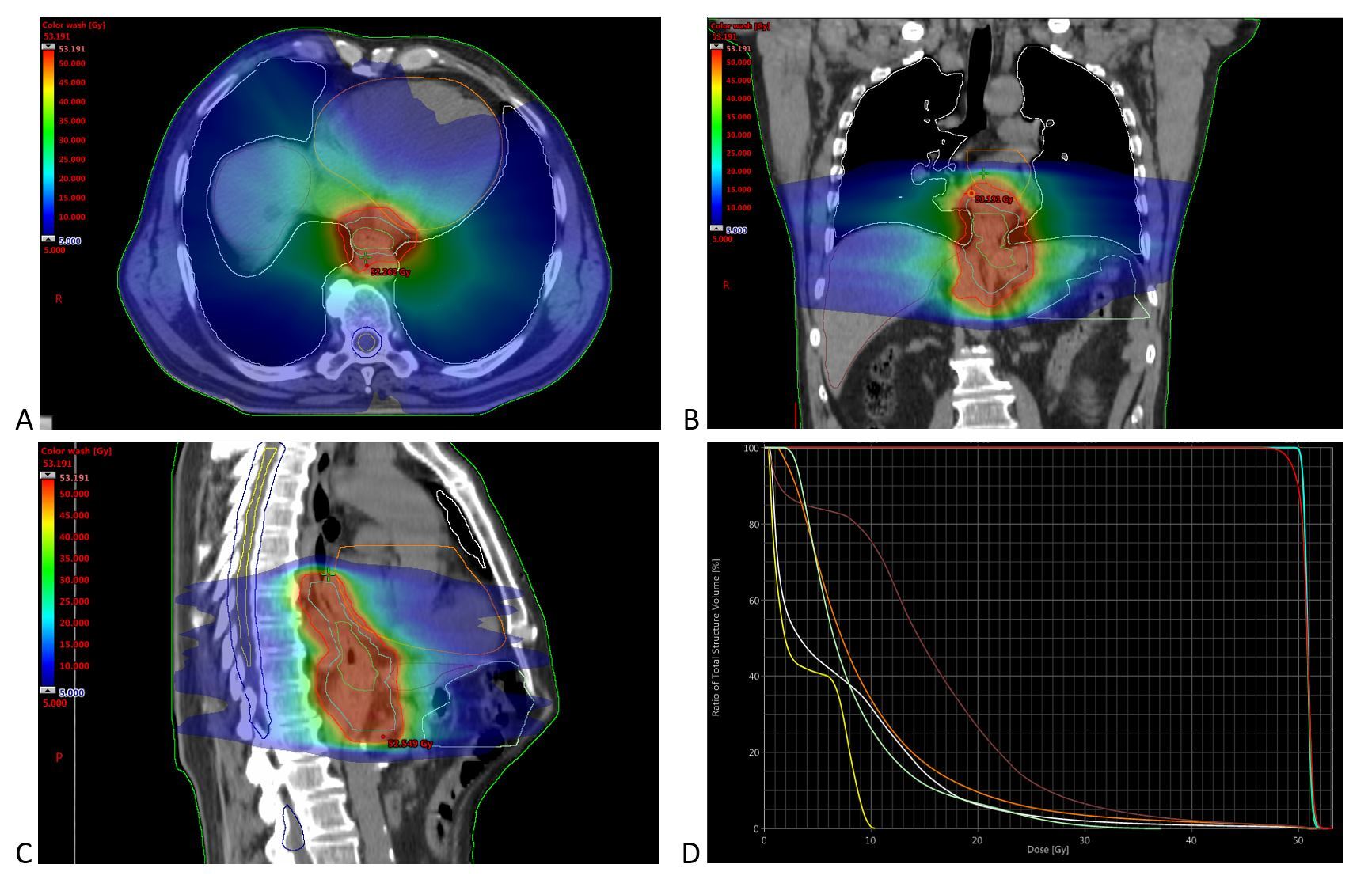


**Figure S2.** Sample plan for an esophageal cancer patient (thoracic esophagus, squamous cell carcinoma, cT3N2) treated with volumetric modulated arc therapy (VMAT). (A) Axial, (B) coronal, (C) sagittal views of the dose distribution, target volumes and organs at risk (OARs). (D) Dose-volume histogram (DVH). The following structures are shown: planning target volume (red); clinical target volume (cyan); gross tumor volume (lemon green); heart (orange); lungs (white); and spinal cord (yellow).


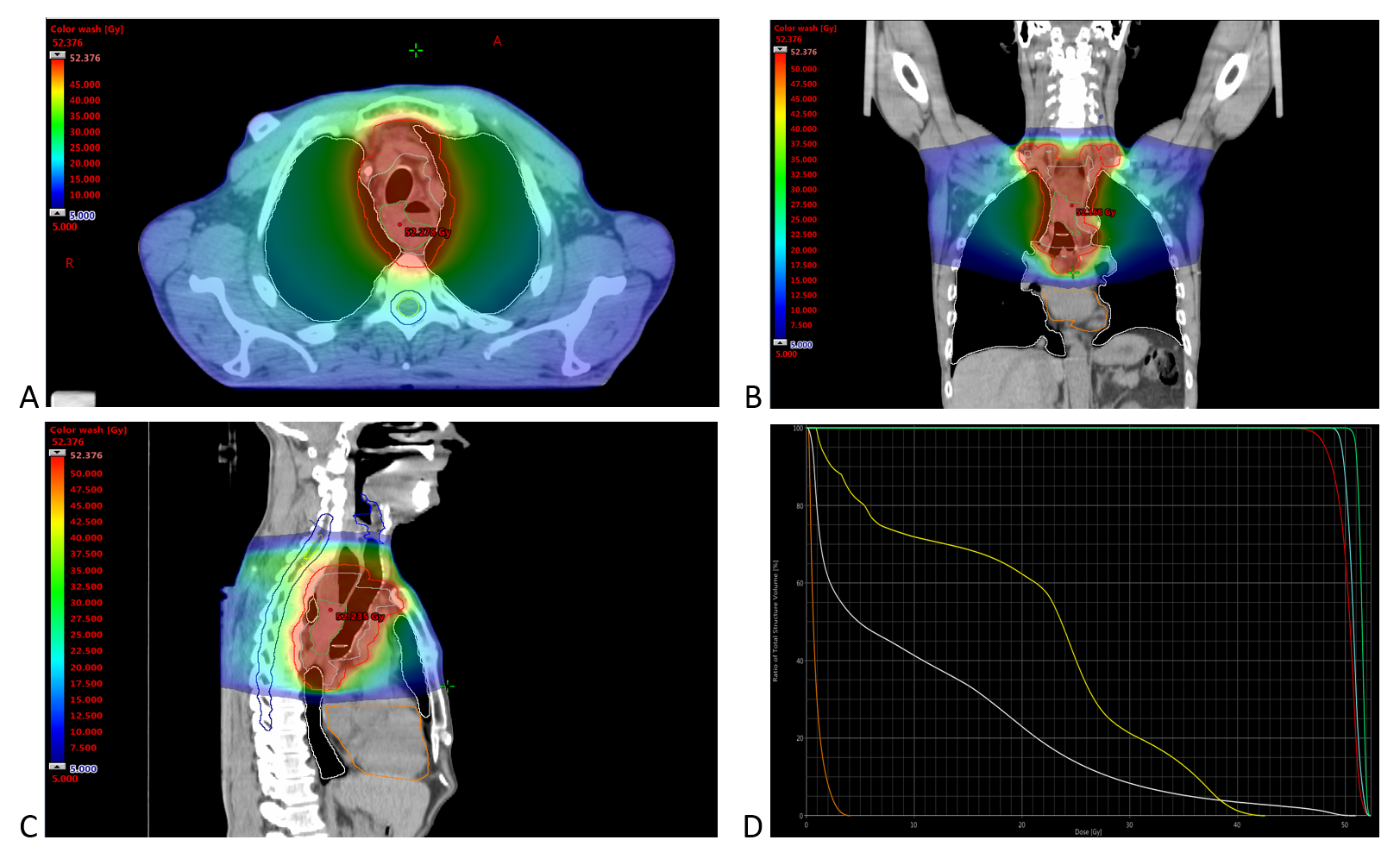

Supplement: Supplementary file 1 [file DataSheet_1.docx]
